# Utilizing vocalizations to gain insight into the affective states of non-human mammals

**DOI:** 10.3389/fvets.2024.1366933

**Published:** 2024-02-16

**Authors:** Jessica C. Whitham, Lance J. Miller

**Affiliations:** Chicago Zoological Society-Brookfield Zoo, Brookfield, IL, United States

**Keywords:** animal welfare, emotion, affective state, vocalization, bioacoustics

## Abstract

This review discusses how welfare scientists can examine vocalizations to gain insight into the affective states of individual animals. In recent years, researchers working in professionally managed settings have recognized the value of monitoring the types, rates, and acoustic structures of calls, which may reflect various aspects of welfare. Fortunately, recent technological advances in the field of bioacoustics allow for vocal activity to be recorded with microphones, hydrophones, and animal-attached devices (e.g., collars), as well as automated call recognition. We consider how vocal behavior can be used as an indicator of affective state, with particular interest in the valence of emotions. While most studies have investigated vocal activity produced in negative contexts (e.g., experiencing pain, social isolation, environmental disturbances), we highlight vocalizations that express positive affective states. For instance, some species produce vocalizations while foraging, playing, engaging in grooming, or interacting affiliatively with conspecifics. This review provides an overview of the evidence that exists for the construct validity of vocal indicators of affective state in non-human mammals. Furthermore, we discuss non-invasive methods that can be utilized to investigate vocal behavior, as well as potential limitations to this line of research. In the future, welfare scientists should attempt to identify reliable, valid species-specific calls that reflect emotional valence, which may be possible by adopting a dimensional approach. The dimensional approach considers both arousal and valence by comparing vocalizations emitted in negative and positive contexts. Ultimately, acoustic activity can be tracked continuously to detect shifts in welfare status or to evaluate the impact of animal transfers, introductions, and changes to the husbandry routine or environment. We encourage welfare scientists to expand their welfare monitoring toolkits by combining vocal activity with other behavioral measures and physiological biomarkers.

## 1 Introduction

Welfare scientists are continually searching for non-invasive, animal-based measures that can be tracked on a regular basis to provide insight into an individual’s welfare status (1–3). Animal welfare is measured on a continuum from poor to good and considers an individual’s mental, physical, and emotional or affective states (2). One way to gain insight into an individual’s inner, affective state is to examine vocal behavior, which can reflect physical, behavioral, and psychological aspects of welfare (4–11). Affective states are emotional experiences that overlap on a spectrum, ranging from fleeting emotions—often triggered by a specific event or object—to longer-term moods (12). Furthermore, it is important to note there are two core dimensions of affect—arousal (intensity) and valence (positive vs. negative). The term *vocalization* is defined here as the active generation of sounds by the vocal tract (pharynx, vocal, nasal and oral cavities, lips and nostrils) that express a distinctive inner state, occurring spontaneously or as the result of an external event (8; Table 1). While this review primarily focuses on vocalizations that are audible to humans, some species also emit ultrasonic or infrasonic calls that may be associated with particular affective states and relevant to welfare status. Vocalizations are valuable for examining the expression of emotions, as sound typically travels well around obstacles, carries long distances, and can change quickly depending on the situation (8, 10, 11, 13). While most studies on non-human animals have investigated the types, rates, and acoustic features of vocalizations emitted in negative contexts, acoustic activity can also serve as an indicator of positive, pleasurable affective states [e.g., (5, 9, 10, 14–18)]. Indeed, Fraser (15) argues that similar to how animals have evolved systems to signal hunger or distress, some species may have evolved to emit signals of positive affect. After all, positive vocalizations can serve a vital communicative function for social, group-living species, by promoting the formation of social bonds and cooperation [e.g., (11, 19–21)].

**Table 1:** Main acoustic parameters and terms discussed in the current article.

Acoustic parameter/term	Definition/Description
Amplitude	Level of energy in a vocalization. Involves the lungs and trachea.
Bandwidth	The difference between the highest and lowest frequency.
Duration	The length of a vocalization from start to finish. Involves the lungs and trachea.
Formants	Frequencies that correspond to the vocal tract’s resonances. Involves the vocal tract (pharynx, vocal/nasal/oral cavities, lips, and nostrils).
Frequency modulation	Variability of the dominant frequency or F0 across the call.
Fundamental frequency (F0)	The lowest frequency in a vocalization. Involves the lungs, trachea, and larynx.
Phonation	The transformation of air flow into sound by vocal fold oscillation.
Spectral noise	Proportion of noise in the vocalization, where the harmonic structure is not clear or cannot be detected.
Vocalization	The active generation of sounds by the vocal tract (pharynx, vocal, nasal and oral cavities, lips and nostrils) that express a distinctive inner state, occurring spontaneously or as the result of an external event.
Vocalization rate	The number of calls that occur per time unit.

Adapted from Briefer (10) and Laurijs et al. (11).

This review focuses on the acoustic activity of non-human mammals. A vast amount of literature exists on human vocal behavior and will primarily be referenced here to gain better insight into the findings for non-human mammals. Across mammals, there are acoustic correlates of the core dimensions of affect—i.e. arousal and valence (10, 12). Overall, when looking across mammalian species, calls increase in the rate of production as arousal increases (10, 22). Affective state can also influence the acoustic features of vocalizations, including the call’s duration, fundamental frequency (i.e., F0, or the lowest frequency of the vocalization), formants (i.e., frequency peaks in the spectrum), and amplitude (10, 22, 23). As arousal increases, the acoustic structure of vocalizations changes in a predictable way across mammalian species, with calls increasing in amplitude and frequency (both F0 and formant-related frequencies) and F0 becoming more variable (10, 23). In other words, as arousal increases, calls are emitted at faster rates and become louder, longer, and harsher (10). However, when considering valence, changes are less consistent across species. In general, a shift in valence tends to be associated with a change in call type (e.g., laughing to crying in humans; whinnies to squeals in horses, *Equus caballus*) (10, 24). Furthermore, calls emitted in positive situations are typically shorter in duration than those that occur in negative contexts (10, 18, 20, 23, 25–31). When the same call type is emitted in both negative and positive contexts, those that occur in positive contexts tend to be shorter in duration but may shift higher or lower in terms of fundamental frequency, depending on the species and/or call of interest [e.g., (10, 18, 28, 32–36)].

This article examines how vocal behavior can provide insight into the affective states of non-human mammals. Specifically, we will:

1. Provide a brief overview of vocal production in non-human mammals.
2. Investigate the construct validity of vocal indicators of affect, with a focus on measures of emotional valence. To review the evidence that vocalizations can be utilized as valid indicators of affective state, we will consider whether vocalizations: (a) reliably vary when individuals experience conditions that are aversive or preferred, (b) reliably vary when individuals experience conditions known to reduce or enhance fitness or survival, (c) are associated with previously validated welfare indicators, and (d) reliably vary when individuals undergo brain stimulation or receive drugs that modulate affect (37). We acknowledge that, at this time, vocalizations are more likely to provide insight into short-term affective states rather than longer-lasting moods.
3. Review methodological considerations and limitations for welfare scientists planning to examine the relationship between acoustic activity and affect.
4. Discuss how the study of vocal behavior can be applied to monitoring the affective states and welfare of animals living under professional care. While the value of tracking vocalizations has been recognized by some welfare scientists working with zoo/aquarium, companion, laboratory, and farm animals, acoustic activity generally has been underutilized in welfare research. We discuss: (a) identifying potential vocalizations of interest by considering a species’ natural history, (b) validating vocal indicators of affect, and (c) incorporating these indicators into welfare monitoring schemes.

## 2 Vocal production

The ability to emit vocalizations relies on the presence of a vocal tract, which in mammals, is characterized by specialized features of both the tracheal tract and pharyngeal cavities (8, 10, 11). According to the source-filter theory (38, 39), the vocalizations emitted by mammals are produced by vibrations of the vocal folds in the larynx (source) and then filtered in the vocal tract (filter). The source determines the fundamental frequency of the call. This aspect of vocal production is influenced by both respiration and phonation (i.e., the transformation of air flow into sound by vocal fold oscillation), thereby involving the lungs and trachea (38). The sound waves generated by the larynx are then filtered by the supralaryngeal vocal tract (filter) (10, 39, 40). The filtering mechanism of the vocal tract—which involves the pharynx, vocal/nasal/oral cavities, lips, and nostrils—shapes the energy distribution of the call and creates the formants by amplifying some frequencies and dampening others (38).

There is evidence that filter-related parameters can provide information about valence (10, 23, 41–44). Indeed, research on humans has shown that filter-related cues vary when comparing emotions that differ in valence but are characterized by similar levels of arousal [e.g., (41, 43, 45, 46)]. As described below, some studies on non-human mammals have examined formants, which may be the key to investigating emotional valence in the future (10, 23, 26, 33, 47). Briefer (10) argues, “it is crucial to measure a large set of parameters including formant frequencies, using the source–filter framework, in order to obtain emotion-specific vocal profiles” (p. 5).

## 3 Evidence of construct validity

There is mounting evidence that vocalizations can be utilized as valid, non-invasive indicators of affective state for non-human mammals. We do not provide a thorough review of the human vocal expression literature here, though studies on human subjects do allow researchers to examine how vocalizations can reliably map onto self-reported affective states (48). For more details on construct validation of vocal indicators of emotions, as well as sensitivity and specificity issues, please see Villain and Briefer (49).

### 3.1 Vocalizations emitted in aversive or preferred contexts

#### 3.1.1 Vocalizations emitted in aversive contexts

Vocalizations emitted in situations that are assumed to be aversive may be indicative of negative affect. Social isolation or separation, which at the very least are considered to be unpleasant for socially-living animals, are associated with changes in acoustic activity for some species [e.g., (5, 50–54)]. In general, mammalian young vocalize frequently when separated from their mother and/or litter-mates [e.g., (5, 50)]. Moreover, numerous studies have demonstrated that a wide range of species emit isolation calls that vary in acoustic structure in relation to various factors (e.g., olfactory, tactile, thermal, early experience, postnatal maternal separation) [e.g., (5, 51–54)]. For instance, Weary et al. (5) discovered that male suckling piglets (*Sus scrofa domesticus*) call repeatedly when isolated from their mother and litter-mates, with those isolated in a cool enclosure vocalizing more often and producing longer, higher frequency calls than those isolated in a warmer enclosure. While these vocalizations appear to be an honest, reliable indicator of need, it can also be assumed that the piglets are experiencing a negative emotional state. Similarly, when being restrained by females who are not their mothers, infant rhesus macaques (*Macaca mulatta*) produce noisy screams, with riskier, severe situations (i.e., longer periods of restraint) being associated with a greater number of calls (55). Finally, in a study of Weddell seal (*Leptonychotes weddellii*) pups, the calls produced by lone pups and those reuniting with their mothers were characterized by longer durations, higher rates of emission, and higher fundamental frequencies than calls emitted during mother-pup contact periods (56). However, these variations in vocal parameters between contexts seem most consistent with the pups’ expression of arousal (10). This is likely the case for many mother-offspring separation studies.

In addition, changes in vocal activity have been reported for adult mammals separated or isolated from conspecifics and are generally considered to be indicative of distress (57–59). In fact, the intensity and frequency of calls may even reflect the strength of the bond between two individuals (58). A study on male cheetah (*Acinonyx jubatus*) pairs found that subjects vocalized at higher rates when separated than during reunions and that sibling pairs vocalized at significantly higher rates than non-siblings (58). Chirps, which exhibited the most individual distinctiveness, were the most common calls produced during separations and only stutters were recorded during reunions. The authors note that in carnivores, short, high-frequency vocalizations with abrupt onset (e.g., the chirp of cheetahs) reflect fear or distress, while low-frequency pulsed and low-amplitude modulated vocalizations (e.g., the stutter of cheetahs) are emitted in affiliative contexts (58; see also 60, 61). Finally, Siebert et al. (62) reported that dwarf goats (*Capra hircus*) emit fewer high bleats but more low bleats when completely isolated, as compared to when they are partially isolated. The authors argue that low bleats may serve a self-calming mechanism and reflect a form of auto-communication. In sum, call type, rate, and even structure may vary when social mammals face separation or isolation from conspecifics.

Alterations in vocal behavior and the acoustic features of calls may also be associated with environmental disturbances. The vocal behavior of farmed silver foxes (*Vulpes vulpes*), who are generally fearful of humans, was impacted by changes in animal-human distance during a human approach test (63). Specifically, the foxes spent an increased proportion of time vocalizing and vocalized at higher frequencies as humans approached. Gogoleva et al. (63) argue that for this species, these variables may, “represent reliable indicators of short-term welfare problems” (p. 8). Alternatively, Castellote and Fossa (64) discovered that the overall vocalization rate (i.e., rate of all vocalization types combined) of beluga whales (*Delphinapterus leucas*) decreased drastically following transportation to new facilities and remained low for four weeks. Notably, food intake was not significantly impacted by the move, and while the whales did display some negative behaviors (e.g., inattentiveness during feeding sessions, low interest/motivation to interact with trainers), these behaviors were not reported by trainers after day eight. Willingness to participate (WtP) in training sessions is an important measure, as a study on bottlenose dolphins (*Tursiops truncatus*) found that WtP was lower in the days leading up to a veterinary diagnosis of a decrease in health state (65). Finally, it should be noted that the overall vocalization rate of the beluga whales studied by Castellote and Fossa (64) also decreased following the introduction of harbour seals (*Phoca vitulina*) and remained low for two weeks. This introduction to the seals occurred approximately four months after the move to the new facility. It is possible that that the acoustic behavior of beluga whales, and specifically the overall production of vocalizations, may be a better welfare indicator than other behaviors (64).

Many studies have investigated the calls emitted by individuals in the context of aggression and social tension. Morton’s (66) “motivational-structural rules” proposed that animals typically emit low-frequency (i.e., low pitch), wide-bandwidth (i.e., noisy) calls in hostile, agonistic contexts and high-frequency (i.e., high pitch), narrow-bandwidth (i.e., tonal) sounds when expressing fear or interacting in a friendly or appeasing manner. Since then, several studies have examined and largely supported these assumptions, though the findings are more consistent for the aggressive contexts [e.g., (67–73)]. In general, vocalizations that occur during agonistic interactions are characterized by long durations, low frequencies, minimal frequency modulations, and wide frequency ranges (reviewed by 10). Some inconsistencies of Morton’s theory may be explained by the fact that both aggression and fear are negatively valenced, while friendly, affiliative behaviors are positively valenced (12, 61). As a result, variations of Morton’s theory have been proposed by other researchers [e.g., (61)].

#### 3.1.2 Vocalizations emitted in preferred/positive contexts

Although it is less common to investigate calls emitted in positive contexts, numerous welfare researchers argue that it is crucial to examine vocalizations that reflect positive affect, as the lack of negative indicators does not imply that an animal is experiencing pleasure or good welfare (3, 9, 15, 74). Indeed, Fraser (15) highlights the value of investigating the noises produced when “all’s well.” Some species’ vocal repertoires include calls that are associated with positive affect. For instance, like humans, orangutans (*Pongo pygmaeus*), bonobos (*Pan paniscus*), chimpanzees (*Pan troglodytes*), gorillas (*Gorilla gorilla*) and siamangs (*Symphalangus syndactylus*) “laugh” when tickled (17). Similarly, Panksepp and Burgdorf (14) suggest that the 50 kHz ultrasonic chirps produced by adolescent rats (*Rattus norvegicus*) while playing and being tickled by experimenters are reminiscent of primitive human laughter (see also 75). In fact, Burgdorf et al. (76) suggest that these emotional vocalizations can serve as “self-report” measures when rats experience positive affective states (20, 75). As noted above, Briefer’s (10) review found that vocalizations emitted in positive contexts tend to be shorter in duration than those that occur in negative contexts, but can vary greatly in fundamental frequency. Indeed, as opposed to the high-frequency ultrasonic vocalizations emitted by rats, some species emit low-frequency calls in positive contexts, including the murmuring of ruminating cows (*Bos taurus taurus*) (77), the coos of infant rhesus macaques (55), and the purrs of gray mouse lemurs (*Microcebus murinus*) receiving grooming from an experimenter (78). Purring and purring-like vocalizations have been reported in the context of relaxed, affiliative interactions (e.g., huddling, mutual grooming, friendly approach) for a wide variety of mammals, including ring-tailed lemurs (*Lemur catta*), northern tree shrews (*Tupaia belangeri*), raccoons (*Procyon lotor*), and various felid species (reviewed by 79). Peters (79) suggests that, in general, purring-like vocalizations, “denote that the vocalizing individual is ‘feeling well’, ‘comfortable’ or ‘content’…” (p. 264). However, it should be noted that purring-like vocalizations have been also reported in animals experiencing pain or distress and may therefore reflect self-soothing, appeasement, or aversive emotional states (79). Ultimately, welfare researchers should specifically attempt to identify vocalizations that are associated with positive events, experiences, and states for the species of interest (see Section 5 for further discussion).

#### 3.1.3 Identifying indicators of valence

**Figure 1:**
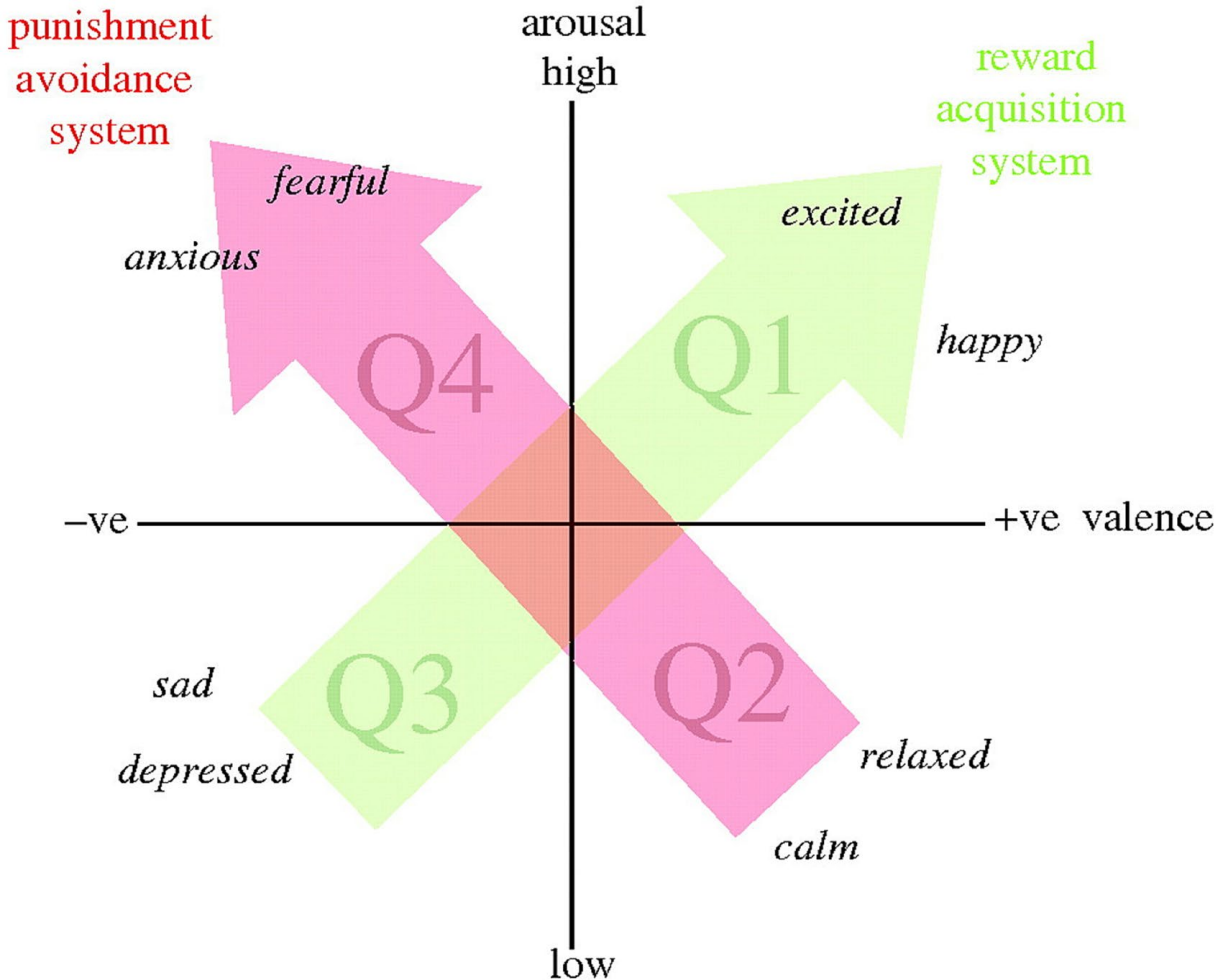
Core affect represented in two-dimensional space. Words in italics indicate possible locations of specific reported affective states (including discrete/basic emotions). Positive affective states are in quadrants Q1 and Q2, and negative states in quadrants Q3 and Q4. Arrows indicate putative biobehavioural systems associated with reward acquisition and the Q3–Q1 axis of core affect (green), and punishment avoidance and the Q2–Q4 axis of core affect (red). Adapted from Russell [e.g., (78)] and Panksepp [e.g., (48)]. Reproduced from Mendl et al. (12).

To identify indicators of valence, some researchers analyze vocalizations produced in *both* positive and negative situations, and in some cases, attempt to control for arousal. This “dimensional approach” proposes that each emotion can be mapped by simultaneously considering both valence and arousal (12, see also 48, 80; Figure 1). For example, vocal indicators of being “relaxed” will be reliably associated with positive valence and low arousal, while indicators of being “anxious” will be associated with negative valence and high arousal. Ultimately, positive affective states will encourage animals to approach stimuli that can promote fitness, while negative affective states trigger avoidance of potentially dangerous stimuli (12).

Most studies that have set out to identify indicators of valence have been conducted on domesticated animals and related species. Briefer et al. (34) identified non-invasive, reliable indicators of both arousal and valence in goats by exposing subjects to four situations (control, anticipating a food reward, food-related frustration, and isolation), for which arousal level could be assessed by measuring heart rate. In positive situations, the goats emitted vocalizations with a lower fundamental frequency range and smaller frequency modulations. In another study that used behavioral and physiological measures to control for arousal, closed-mouth grunts produced by adult pigs during a positive situation (access to food/toys while paired with a conspecific) varied from those emitted during a negative situation (social isolation) in various ways, including shorter durations, differences in formant-related parameters (see below), and lower fundamental frequencies (23). Similar results were found for wild boars (*Sus scrofa*), with subjects emitting shorter, lower frequency vocalizations in positive situations (food reward or affiliative contexts) than in negative situations (agonistic encounters), when controlling for arousal (30). In a study of horse whinnies, those produced in a positive context (reunion with group members) were found to be shorter in duration than whinnies emitted in a negative context (separation from group members) (28). Furthermore, two fundamental frequencies, F0 (the lower fundamental frequency) and G0 (the higher fundamental frequency), were discovered, with the latter encoding valence by being lower in positive situations. Interestingly, while whinny duration and G0 frequency were not a reliable indicator of valence in Przewalski’s horses (*Equus przewalskii*): (1) positive and negative contexts were associated with particular call types and (2) acoustic structure varied according to valence (81). Overall, however, there is evidence that vocalizations emitted in positive contexts are generally shorter in duration with lower fundamental frequencies (10, 23).

The findings for domestic dogs (*Canis familiaris*) break from the pattern of calls having lower fundamental frequencies when emitted in positive versus negative contexts. For instance, in a study of dog barks, those produced in the context of play (and isolation) had a different acoustic structure—shorter inter-call intervals, shorter durations, higher frequencies, and more pitch and amplitude modulation —than barks emitted during “a disturbance situation” (stranger ringing the doorbell) (82). Similarly, Faragó et al. (26) reported that growls were characterized by shorter durations and higher fundamental frequencies in the context of play than in negative situations (exposure to a threatening stranger, food guarding) (26). On the contrary, a separate study of dog growls found that while growls produced in the context of play (playing with owner) were shorter than those emitted during the context of aggression (an approaching stranger), they did not vary in fundamental or formant frequencies (18). As noted earlier, when comparing the same call type across negative and positive situations for a particular species, the vocalizations produced in positive contexts may shift to a higher frequency (10, 23).

It is less common for studies investigating emotional valence to be conducted on non-domesticated species. In African elephants (*Loxodonta africana*), the rumbles produced by low-ranking females in calm social contexts differed from those emitted while interacting with dominant elephants, with the former having lower and less variable fundamental frequencies, as well as lower amplitudes and shorter durations (32). In a follow-up study, Soltis et al. (33) attempted to take a dimensional approach and control for arousal by examining rumbles in the following contexts: high intensity/negative social context (dominance interactions), high intensity/positive social context (affiliative interactions), and a low intensity/neutral social context. The authors found that females produced calls with longer durations, as well as higher and more variable fundamental frequencies and amplitudes, when comparing the negative context to the neutral context. However, rumbles emitted in the positive context were similar in duration to the negative context, and when considering most of the acoustic features (e.g., F0 range, amplitude range, max amplitude), the findings were intermediate between the neutral and negative contexts. Soltis et al. (33) concluded that the results were most consistent with rumbles reflecting affect intensity (regardless of valence), with the acoustic responses in the positive context signaling an intermediate level of arousal. However, they suggested that the combination of acoustic features may create a “vocal signature” of valence. After all, while the features that increase in both positive and negative contexts may reflect arousal, those that only increase in the negative context may reflect valence. A study of farmed spotted paca (*Cuniculus paca*) found that certain acoustic parameters of snorts, barks, and roars varied according to the valence of situation, and that snorts were more likely to be emitted in a negative situation (enclosure cleaning) than a positive situation (feeding time) (83). Finally, in a study of bonobo peep vocalizations, the acoustic structure of peeps produced during positive situations (feeding) could not be distinguished from those emitted during neutral situations (travel, rest) (35). However, peeps produced in negative situations (agonism and alarm) were characterized by shorter durations and higher mean fundamental frequencies.

Finally, just as research on humans has shown that formants may vary with valence, formant-related parameters may be valuable to the study of non-human animal emotions (10). Relatively few studies have examined formant-related features, and so far, the results are inconsistent. In Briefer et al’s. (23) study of pigs’ closed-mouth grunts, calls produced in the positive situation (access to food/toys while paired with a conspecific) were characterized by higher formants and a smaller range of the third formant than grunts emitted in the negative situation (social isolation). A study of feral cat (*Felis catus*) meows reported that the first formant of calls was higher in a positive context (affiliation) than a negative context (agonism) (47). Alternatively, for African elephants, rumbles produced in a negative social context (dominance interactions) had higher first formants than those emitted in a neutral context (33). For dogs, growls emitted in a positive context (play) were characterized by lower formant dispersion—which is suggestive of lower formants—than those produced in a negative context (exposure to a threatening stranger, food guarding) (26). As noted above, however, Taylor et al. (18) did not find differences in formant-related parameters when comparing dog growls produced in positive and negative contexts (play vs. aggression). Other studies described in this section, including the studies of horse whinnies (28) and goat calls (34), reported no significant effects of valence on formants. Further research is needed, as results may be influenced by the use of different species, contexts (e.g., play vs. affiliation for positive contexts, isolation vs. agonism for negative contexts), arousal levels, etc. (23).

### 3.2 Vocalizations emitted in situations that reduce or enhance fitness or survival

Numerous studies have investigated the calls emitted by animals facing situations that have the potential to reduce fitness or survival. There is evidence that vocalizations can be honest indicators of pain, which is assumed to be associated with negative affect and poor welfare [e.g., (84, 85)]. In general, animals experiencing severe pain emit calls as a high rate (84–87). For example, several studies have found that male piglets undergoing castration produce calls at high rates, with these vocalizations generally having high frequencies, amplitudes, and durations (85, 86, 88, 89). Furthermore, the calls emitted by piglets experiencing pain distress can be distinguished from vocalizations produced during other types of distress (i.e., cold and hunger) (90).

An extensive amount of research has been conducted on calls produced in fear-inducing situations, such as the alarm calls emitted in the presence of predators (91, 92). It is important to note that while some species emit alarm calls frequently when facing predators or other potential threats, others may become less vocal or even silent (92, 93). For example, when both free-ranging and professionally-managed beluga whales (*Delphinapterus leucas*) are exposed to predators or noise disturbances (e.g., killer whales, boat engines), their acoustic activity may decrease or cease completely (64, 94–96). Similar results have been found for free-ranging narwhals (*Monodon monoceros*), suggesting that it may be adaptive for marine mammals to reduce or eliminate vocal activity when encountering potential threats (97). Indeed, it can be advantageous for frightened, startled, or threatened individuals to avoid detection in the presence of predators by remaining silent (64, 94). It is vital to investigate the evolution and natural history of specific vocalizations, as well as whether the call may have co-evolved with other behaviors (e.g., hiding vs. freezing) (4, 98). Section 5 further discusses the importance of understanding the natural history of a species’ vocal repertoire.

### 3.3 Vocalizations associated with previously validated welfare indicators

Vocalizations produced in both negative and positive contexts are often temporally associated with previously validated physiological or behavioral welfare indicators (reviewed by 10). While researchers assessing the arousal and valence of calls should attempt to incorporate physiological indicators, relatively few studies have integrated biomarkers such as heart rate, respiration, adrenaline, or cortisol/corticosterone (50, 59, 99, 100; reviewed by 10). Overall, however, most evidence seems to point to physiological indicators being associated with indicators of emotional arousal (e.g., 34, 99; reviewed by 10).

Some studies have examined how vocal behavior is associated with cardiac activity or respiration rates. In Briefer et al’s. (34) study of goat vocalizations, it was discovered that heart rate variability was not influenced by valence but was impacted by arousal, with high arousal situations being associated with lower heart rate variability and higher respiration rates (see also 101). The authors noted that they did not identify a good physiological indicator of valence. A similar conclusion was drawn for gilts participating in a standard human approach test. Specifically, gilts that squealed more not only displayed more locomotor behavior and interacted more with humans, they also had higher mean heart rates and lower heart rate rise in response to human touch (99). The authors argued that these findings were indicative of higher arousal levels.

Other researchers have investigated the relationship between vocal behavior and hypothalamic–pituitary–adrenal (HPA) axis activity. Boinski et al. (102) discovered that the group mean of terrestrial predator alarms (TPA) for singly housed adult male brown capuchins (*Cebus apella*) was positively correlated with mean group levels of fecal cortisol, as well as abnormal behaviors. Furthermore, it should be noted that those housed in an enriched environment (i.e., cages with toys and foraging boxes) had a lower mean TPA rate in response to humans than the control group (i.e., cages with only a plastic chain). As a result, the authors suggested that individuals housed under low enrichment conditions were more “stressed” and reactive and that TPAs can serve as a “first-line” indicator of welfare. In an experimental study that involved separating adult pigs from groupmates, it was determined that increasing rates of squeal-grunts were positively associated with plasma levels of adrenaline, while rates of grunts were inversely associated with cortisol levels (103). However, it should be noted that within these call types, acoustic parameters were not significantly correlated with either hormone. While laboratory-housed adult marmosets (*Callithrix jacchus*) produced high levels of phee calls when separated from groupmates and placed in a novel environment for 20 min, there was no association between the number of calls emitted and cortisol levels (59). The results from infant separation studies are also mixed. For instance, in a 2-wk maternal separation study involving infant bonnet macaques (*Macaca radiate*) and pigtail macaques (*Macaca nemestrina*), mean plasma free and total cortisol were positively associated with distress vocalizations and slouching and negatively associated with play during the first week of separation (104). However, in a study that examined the separation calls of infant squirrel monkeys (*Saimiri sciureus*) over a 24-h period, the intensity of calling was not predictive of cortisol levels (105). Clearly, such studies vary greatly in terms of the species of interest, age of the subjects, and methodology, including the length of the separation (hours vs. weeks).

If possible, researchers should attempt to combine measures of heart rate, HPA activity, and behavior. In a study that examined the responses of ewes (*Ovis aries*) separated from their lambs, ewes exhibited a significant increase in activity, vigilance, bleats, heart rate, and cortisol levels (100). Furthermore, these behavioral and physiological responses were correlated with changes in the ewes’ voice characteristics, including an increase in total duration, energy, and fundamental frequency. The authors argue that these behavioral, physiological, and acoustic changes reflect negative emotional states and that certain bleat characteristics may serve as markers of distress.

### 3.4 Insights from brain stimulation studies and pharmacological research

Numerous studies have investigated the neural substrates underlying vocal behavior. The amygdala, which is involved in the expression of both negative and positive emotions in mammals, plays a role in vocal production (106; reviewed by 11). Furthermore, there is evidence that the various call types comprising a species’ vocal repertoire are generated via specific pathways that begin in the amygdala (11, 106, 107). Indeed, Jürgens (108) was able to induce vocalizations in squirrel monkeys by electrically stimulating the anterior cingulate cortex, which receives input from the amygdaloid complex. The amygdaloid complex not only mediates certain emotions (e.g., fear, anxiety) but also regulates the HPA axis (109–111). Studies have shown that different circuitries are involved with calls linked to negative states versus positive states (20, 112). For instance, the two types of acoustically distinct ultrasonic calls produced by rats—the 22 kHz vocalizations produced in negative contexts and the 50 kHz calls produced in positive contexts—are linked to the neural substrates associated with the generation of negative and positive states (20, 113). Specifically, electrical activation of the mesolimbic cholinergic system induces a negative emotional state and the production of 22 kHz calls, while the activation of the mesolimbic dopaminergic system induces a positive emotional state and 50 kHz calls (see also 114, 115). Briefer (10) notes that specific brain circuits responsible for emotions have been linked to particular vocalizations in other species. If specific vocalizations can be induced (or inhibited) by stimulating (or lesioning) particular parts of the brain, and if those parts of the brain mediate certain affective states, evidence exists for construct validity.

Vocalizations can also be induced (or inhibited) by administering drugs that impact brain circuits associated with emotional states [e.g., (116–118)]. In pigs, an increased vocalization rate and activity can be induced by centrally injecting subjects with anxiogenic peptides, such as corticotropin releasing hormone (117, 119). Alternatively, when female rhesus macaques were administered metyrapone to suppress cortisol production, subjects emitted significantly fewer alarm calls in response to their infants being threatened, as compared to controls (120). A series of studies reported that the 50 kHz ultrasonic vocalizations of rats increased in response to the administration of euphorigenic drugs, while sickness-inducing doses of lithium chloride decreased the number of these calls [e.g., (115, 121)]. While these invasive studies are not recommended for species living in certain settings (e.g., zoos, aquariums, shelters, and sanctuaries), and there are obvious ethical concerns, this line of research has informed studies of vocal behavior by highlighting the links between specific brain circuits, affective states, and vocalizations.

## 4 Methodological considerations and limitations

For those planning to integrate measures of vocal behavior into studies aimed at assessing the affective states of individual animals, several methodological considerations and limitations must be addressed. While not always realistic for animals living in certain professionally managed settings (e.g., zoos, wildlife sanctuaries), the most informative studies will incorporate: (1) spectrographic analyses to identify links between specific acoustic features and particular affective states and (2) experiments that adopt a dimensional approach (i.e., that examine vocal behavior in both positive and negative situations of similar arousal) (12). Indeed, when assessing and monitoring affect, the goal is to identify indicators of valence—not just arousal—by pinpointing which acoustic features are associated with negative versus positive contexts.

In some cases, it may only be feasible to examine vocalization rates. Depending on the species of interest, tracking the rate of combined vocalizations or of specific call types may provide useful information. Even for researchers who are unable to purchase recording equipment and can only conduct behavioral observations, it is possible to establish baseline vocalization rates for individual animals and to track this measure across various contexts. As described above, some species become less vocal or even silent when facing threats or environmental disturbances [e.g., (64)]. Animals experiencing severe pain, extreme lethargy, or learned helplessness—states likely associated with negative affect—may also remain silent. Furthermore, it is important to recognize that individuals of the same species may vary in terms of how vocal they are in particular contexts, which may be due to differences in genetics, temperament, and early experiences [e.g., (122–124)]. Finally, it is crucial to remember that some species produce ultrasonic or infrasonic vocalizations, which would be missed if not recorded with the proper equipment calibrated to detect the appropriate frequency range (125).

Fortunately, researchers and animal management staff who have the means to purchase recording equipment and software are able to take advantage of recent technological advances in the field of bioacoustics. Modern acoustic monitoring systems make it possible for researchers to continuously track individuals under a variety of circumstances (e.g., in the dark, underwater, after-hours), even for group-housed or nocturnal animals (126, 127). Before initiating a study, a considerable amount of effort must be invested in weighing options for recording and analyzing calls. Vocalizations can be recorded by introducing hydrophones or microphones to enclosures or by utilizing animal-attached devices (e.g., collars) (74). Clearly, characteristics of the species of interest (e.g., size, physical attributes, ability of the wearer/conspecifics to manipulate the device) will influence the type of recording device that is chosen. If animal-attached devices are not feasible, the researcher will have to determine whether to employ directional or omnidirectional microphones. Certain settings are associated with particular challenges when utilizing recording devices. For example, in zoos, visitors contribute noise to the environment, and certain surfaces (e.g., windows, glass panels) reflect sound or even mask calls (128). For enclosures with a water feature, the air-water interface can reflect and reverberate sounds (128). For species that spend most or all of their time underwater, life support systems may produce additional noise that may not only interfere with recordings but also influence vocal behavior (62). Finally, low frequencies, which can travel longer distances and are less likely to be impacted by dense vegetation, are more likely to be captured by microphones (128–130). As a result, careful consideration must be given to the placement of microphones in the enclosure. Schneider and Dierkes (128) recommend taking the height of the enclosure into account and localizing in three dimensions, though this can be difficult if the enclosure is uneven in height. These researchers advise using least four microphones for two-dimensional localization of a vocalization but caution that even more are necessary in large enclosures.

Recent advances in bioacoustics software allow for continuous monitoring, automatic detection of calls, and real-time sound analyses. For instance, Schneider and Dierkes (128) tested the LASER sound localization software, which can accurately estimate the position of the animal that is vocalizing, thereby allowing the call to be assigned to the correct subject. Even when considering otters, which move quickly and closely together in an aquatic environment, 78% of the calls could be assigned to the correct caller. Similarly, the National Marine Mammal Foundation’s Welfare Acoustic Monitoring System (WAMS) employs hydrophones to continuously capture, count, and localize vocalizations, as well as specialized software that automatically compares the current data to historical output/baseline data. In fact, this real-time alert system includes an alarm module that triggers an email alert (complete with a call count, screenshot of the spectrogram, and localization information) if the call rate surpasses the user-defined threshold (127, 131). For farm animals, Briefer et al. (29) demonstrated that an automated recognition system allowed for real-time discrimination of valence, as well as the context of call production. The researchers assessed two methods for call classification in this study: (1) an image classification neural network (i.e., a machine learning model that can recognize patterns in images) based on spectrograms of calls and (2) a permuted discriminant functional analysis (i.e., a multivariate statistical method used in bioacoustics research to distinguish calls) based on selected vocal parameters—with the former having higher classification accuracy. The authors concluded that this automated emotion monitoring tool can ultimately be used to track welfare on farms. A thorough discussion of automated acoustic monitoring, advanced computational audio analysis methods, and spectrographic analyses is beyond the scope of this paper and can be reviewed elsewhere (8, 40, 126).

## 5 Discussion

For researchers interested in assessing or monitoring the affective states of individual animals, integrating measures of vocal behavior can be extremely valuable. When initiating a study to investigate associations between acoustic activity and affect, the first step is to identify potential calls of interest by examining the natural history of the species’ vocal repertoire (4). This can be accomplished by conducting preliminary observations and reviewing the literature (4, 64). Specifically, the researcher can better understand the role particular vocalizations play in the species’ behavioral repertoire by examining the call from a developmental and evolutionary perspective (4). For example, a given vocalization may only be produced by specific age/sex classes or may have co-evolved and be temporally associated with other behaviors (98). While reviewing the literature, the researcher should determine whether there is evidence that the species of interest emits ultrasonic or infrasonic vocalizations, which cannot be perceived by humans. If feasible, the researcher can collect recordings from individuals of various age/sex classes and conduct spectrographic analyses to identify all vocalization types. Ultimately, studies may be limited to investigating vocalizations that are audible to humans, due to the prohibitive costs of recording equipment and/or other practical issues (e.g., facility type, presence of visitors). Once potential calls of interest have been identified, the challenge is to highlight indicators of valence, not just arousal. Briefer (10) explains that this can be challenging because research on valence should compare calls emitted in both negative and positive contexts that are characterized by similar levels of arousal, and this can be difficult to find due to the fact that expressions of negative affect are typically more intense (see also 9).

It has become increasingly common for researchers to adopt a dimensional approach. As Briefer (10) notes, “this approach is useful for the study of animal emotions because it allows researchers to investigate differences between emotional states of low versus high arousal and of positive versus negative valence, without having to infer the specific emotion that the animal is experiencing” (p. 4). As described earlier, researchers can assess arousal level by integrating physiological biomarkers, such as measures of heart rate and respiration. Whitham and Miller (74) discuss technology and equipment that can be utilized to non-invasively assess autonomic nervous system (ANS) activity to provide insight into physiological functioning and arousal level. Ultimately, the goal is to highlight indicators of valence—whether they be particular call types or acoustic features.

Previous studies on both human and non-human mammals have provided evidence of vocal correlates of valence. When summarizing the literature for non-human mammals, there is growing evidence that: (1) a shift in valence is associated with a change in call type, (2) vocalizations emitted in positive contexts tend to be shorter in duration, and (3) fundamental frequencies may shift lower or higher when a particular call type is emitted in both positive and negative situations (10). This means that, for a given study species, researchers should aim to determine whether: (1) specific call types are more likely to occur (or exclusively occur) in certain contexts, (2) call duration varies when comparing vocalizations produced in negative versus positive contexts, and (3) calls of interest that are produced in both negative and positive contexts vary in terms of acoustic structure. Therefore, as a first step, a concerted effort should be made to analyze calls produced during situations/events known to be positive or pleasurable, as well those known to be negative or aversive. For example, are certain vocalizations more likely to be produced while feeding, playing, or receiving grooming? Are other vocalizations more likely to occur while initiating/receiving threats or aggression? Does call production vary by age/sex? Even if the researcher does not have the ability to analyze the duration or acoustic structure of calls, it should be possible to determine whether call types and rates vary across contexts. If the resources are available, researchers also can examine whether other parameters might be indicative of valance for the species of interest. Indeed, research on humans has demonstrated that vocalizations associated with positive affect tend to be characterized by certain features including, narrower frequency ranges, lower amplitudes, higher formants, less spectral noise, and an earlier position of maximum peak frequency (43, 45, 46, 132; reviewed by 10).

Overall, there is great potential for using vocal behavior to assess and monitor the emotions of individual animals. Vocalizations are well-suited for investigating the expression of an animal’s inner state. As opposed to facial expressions or most behavioral states, vocalizations can communicate information to numerous individuals simultaneously, even if they are not in close proximity. Indeed, sound generally travels around features in the environment and carries long distances, though the features of certain settings (e.g., zoo enclosures with glass panes or lush vegetation) may present challenges when studying acoustic activity (8, 10, 11, 13, 128). Fortunately, such challenges can be overcome by introducing multiple microphones into the enclosure and arranging them strategically. Another benefit to studying vocal behavior is that call types and acoustic structure can change quickly to accurately reflect the caller’s current state. While this means that many vocalizations can serve as honest, reliable indicators of short-term emotions, vocal activity may not be as helpful for gaining insight into an individual’s long-term affective states or mood. Vocal behavior can still be a valuable indicator. For instance, if baseline data are available, vocalizations can be: (1) monitored to evaluate responses to changes in the environment and/or routine, or (2) tracked regularly to proactively highlight potential shifts in welfare status.

Indeed, vocal activity is an ideal measure to be integrated into welfare monitoring schemes for animals living under professional care, as vocal indicators can be tracked continuously and non-invasively. For some species, vocalizations may even be a better welfare indicator than some traditional measures, as changes in vocal activity (e.g., rates of production) may last longer than changes in appetite or other behaviors (64). Jones et al. (131) note that acoustic activity is underutilized in welfare research—particularly for aquatic animals—and promote the use of acoustic monitoring systems (e.g., WAMS). Real-time systems can trigger an alert, after which changes in call rates (for the group or individuals) can be compared to data from veterinary exams and behavioral observations. The authors note that these sorts of systems have the potential to: (1) detect instances of aggression, early signs of illness, and anthropogenic/environmental sound disturbances and (2) determine whether a certain level of “communicative chatter” may serve as, “a positive signal of the ‘status quo’” (p. 231). Given recent technological advances, call identification algorithms can even be applied to detect certain types of vocalizations (e.g., distress calls) or the real-time discrimination of valence (29, 126). Of course, even if researchers are unable to utilize recording equipment or bioacoustics software, ongoing behavioral monitoring can be conducted. Ultimately, animal care professionals can examine vocal behavior (even simply call rates) to highlight potential welfare issues and to proactively intervene by introducing changes to the environment and/or routine.

Finally, one of the most appealing reasons for integrating acoustic vocal behavior into studies of affect is that vocalizations allow for an emphasis on positive welfare. Indeed, while welfare studies traditionally have focused on negative indicators, the presence of positive affective states may be more relevant to welfare assessments than the absence of negative affective states (3, 9). Fortunately, many species’ vocal repertories include calls that are associated with positive affect—from laughing gorillas to chirping rats to purring cheetahs (10, 79). We also recommend that welfare researchers investigate the vocalizations emitted by individuals facing challenges (e.g., novel enrichment such as puzzle feeders) designed to provide stimulation and promote natural behaviors (e.g., exploration, object manipulation). The vocalizations emitted by individuals in these situations—which are assumed to be stimulating and beneficial—may allow us to gain insight into how animals respond to eustress and short-term stressors.

Ultimately, the goal is to identify reliable, valid indicators of emotional valence for the species of interest—whether they be particular call types, vocalization rates, and/or acoustic features—and to integrate these with behavioral and physiological welfare measures. Vocal activity can then be monitored for individual animals continuously across various contexts and to evaluate responses to both acute and chronic stressors. A comprehensive welfare monitoring toolkit allows for researchers to not only conduct baseline monitoring but to also evaluate the impact of animal introductions, transfers, and changes to the husbandry routine or environment.

## 6 Conclusion

While the field of animal welfare science continues to expand, there is a need to identify indicators of affective state, and especially emotional valance, for most species. The human literature, as well as studies on various non-human mammals, have demonstrated that vocalizations can serve as valid indicators of short-term affect. Researchers working in zoological facilities, agricultural settings, companion animal shelters, and wildlife sanctuaries could greatly benefit by integrating measures of acoustic activity (e.g., vocalization rate, duration, acoustic structure) into systematic welfare studies, as well as ongoing monitoring schemes for individual animals. Having the ability to detect changes in vocal indicators of valence would allow welfare scientists to intervene when an individual’s welfare seems compromised and to make informed management decisions. Incorporating measures of positive affect is vital, as the lack of negative behaviors alone does not imply that an individual is experiencing pleasurable states [e.g., (9)].

Ultimately, combining vocal indicators of affect with traditional welfare measures can help researchers conduct more comprehensive assessments of individual animal welfare, and in particular, will allow for a focus on positive welfare. Other indicators that can be incorporated in this toolkit include physiological biomarkers of welfare (e.g., measures of cardiac activity, cortisol/corticosterone) and various behavioral states and events. In the future, acoustic activity could be monitored continuously to detect shifts in welfare status or to assess the effects of animal transfers, introductions, and changes to the husbandry routine or environment.

## Author contributions

JW: Conceptualization, Writing – original draft, Writing – review & editing. LM: Conceptualization, Writing – review & editing.
