# Corrigendum: Utilizing vocalizations to gain insight into the affective states of non-human mammals

**DOI:** 10.3389/fvets.2024.1404906

**Published:** 2024-04-04

**Authors:** Jessica C. Whitham, Lance J. Miller

**Affiliations:** Chicago Zoological Society-Brookfield Zoo, Brookfield, IL, United States

**Keywords:** animal welfare, emotion, affective state, vocalization, bioacoustics

In the published article there was a citation missing in Introduction, page 2, paragraph 3.

The section previously stated:

“2) Investigate the construct validity of vocal indicators of affect, with a focus on measures of emotional valence. To review the evidence that vocalizations can be utilized as valid indicators of affective state, we will consider whether vocalizations: (a) reliably vary when individuals experience conditions that are aversive or preferred, (b) reliably vary when individuals experience conditions known to reduce or enhance fitness or survival, (c) are associated with previously validated welfare indicators, and (d) reliably vary when individuals undergo brain stimulation or receive drugs that modulate affect. We acknowledge that, at this time, vocalizations are more likely to provide insight into short-term affective states rather than longer-lasting moods.”

The corrected section is below:

“2) Investigate the construct validity of vocal indicators of affect, with a focus on measures of emotional valence. To review the evidence that vocalizations can be utilized as valid indicators of affective state, we will consider whether vocalizations: (a) reliably vary when individuals experience conditions that are aversive or preferred, (b) reliably vary when individuals experience conditions known to reduce or enhance fitness or survival, (c) are associated with previously validated welfare indicators, and (d) reliably vary when individuals undergo brain stimulation or receive drugs that modulate affect (37). We acknowledge that, at this time, vocalizations are more likely to provide insight into short-term affective states rather than longer-lasting moods.”

In the published article there was a citation missing in Evidence of construct validity, page 3.

The section previously stated:

“There is mounting evidence that vocalizations can be utilized as valid, non-invasive indicators of affective state for non-human mammals. We do not provide a thorough review of the human vocal expression literature here, though studies on human subjects do allow researchers to examine how vocalizations can reliably map onto self-reported affective states (47).”

The corrected section is below:

“There is mounting evidence that vocalizations can be utilized as valid, non-invasive indicators of affective state for non-human mammals. We do not provide a thorough review of the human vocal expression literature here, though studies on human subjects do allow researchers to examine how vocalizations can reliably map onto self-reported affective states (48). For more details on construct validation of vocal indicators of emotions, as well as sensitivity and specificity issues, please see Villain and Briefer (49).”

The authors apologize for this error and state that this does not change the scientific conclusions of the article in any way. The original article has been updated.
